# Brain Network Studies in Chronic Disorders of Consciousness: Advances and Perspectives

**DOI:** 10.1007/s12264-018-0243-5

**Published:** 2018-06-18

**Authors:** Ming Song, Yujin Zhang, Yue Cui, Yi Yang, Tianzi Jiang

**Affiliations:** 1 National Laboratory of Pattern Recognition, Institute of Automation, The Chinese Academy of Sciences, Beijing, 100190 China; 2 Brainnetome Center, Institute of Automation, The Chinese Academy of Sciences, Beijing, 100190 China; 3 University of Chinese Academy of Sciences, Beijing, 100190 China; 4 Department of Neurosurgery, PLA Army General Hospital, Beijing, 100700 China; 5 CAS Center for Excellence in Brain Science and Intelligence Technology, Chinese Academy of Sciences, Beijing, 100190 China; 6 Key Laboratory for Neuroinformation of the Ministry of Education, School of Life Science and Technology, University of Electronic Science and Technology of China, Chengdu, 625014 China; 7 The Queensland Brain Institute, University of Queensland, Brisbane, QLD 4072 Australia

**Keywords:** Disorders of consciousness, Neuroimaging, Brain network, Brainnetome

## Abstract

Neuroimaging has opened new opportunities to study the neural correlates of consciousness, and provided additional information concerning diagnosis, prognosis, and therapeutic interventions in patients with disorders of consciousness. Here, we aim to review neuroimaging studies in chronic disorders of consciousness from the viewpoint of the brain network, focusing on positron emission tomography, functional MRI, functional near-infrared spectroscopy, electrophysiology, and diffusion MRI. To accelerate basic research on disorders of consciousness and provide a panoramic view of unconsciousness, we propose that it is urgent to integrate different techniques at various spatiotemporal scales, and to merge fragmented findings into a uniform “Brainnetome” (Brain-net-ome) research framework.

## Introduction

Recent progress in intensive care has increased the survival of patients with severe brain damage. Some can recover consciousness from an acute brain insult, while some can tragically fall into chronic disorders of consciousness (DOCs). These DOC patients are stable but disabled and bedridden, unable to speak or signal their thoughts and intentions. Their lives need laborious care. With proper nursing care to avoid bedsores and infections, these patients can survive for years. Therefore, the social, economic, and ethical consequences associated with chronic DOCs are tremendous [1].

Management of a chronic DOC patient requires carefully reaching the correct diagnosis, pronouncing an evidence-based prognosis, and thoughtfully evaluating any medical interference. To date, these clinical assessments still depend on expert observation of the patient’s behavior over a sufficient period of time. Taking the diagnosis as an example, DOC patients can be classified into distinct diagnostic entities according to the surviving consciousness level [2]. Specifically, patients in a vegetative state or unresponsive wakefulness syndrome (VS/UWS) can retain an irregular but cyclic state of circadian sleeping and waking unaccompanied by any behaviorally detectable expression of awareness of themselves or recognition of external stimuli. When patients show fluctuating but reproducible behavioral signs of awareness but remain unable to functionally communicate or use objects, they are considered to be in a minimally conscious state (MCS). The Coma Recovery Scale - Revised (CRS-R) is now the gold standard for diagnosis to distinguish VS/UWS from MCS and conscious (exit-MCS) patients. In spite of its unquestionable value, an increasingly popular proposal is challenging this paradigm based solely on behavior [3]. On the one hand, a patient’s motor impairment, sensory deficit, cognitive damage, fluctuation of vigilance, and medical complications can give rise to misjudgments; on the other hand, for the assessor, a lack of knowledge regarding DOCs, poor training, and non-use of adequate behavioral scales are additional elements that contribute to a high possibility of mistakes. Consequently, careful and repeated behavioral assessments are considered to be particularly important for a precise diagnostic and prognostic judgment [4]. However, behavioral assessments are inevitably subjective and vulnerable to a variety of personal influences [5]. Physicians and scientists have therefore been seeking accurate and objective markers for assessing the level of consciousness of a DOC patient [6, 7].

The human brain comprises about one hundred billion neurons, with thousands of trillions of connections between them. Its complexity is not only reflected in the numbers of neurons and connections, but also by how the brain is wired on different scales and how such patterns of connections produce functions, including consciousness. More and more studies suggest that the human brain can be studied as hierarchical complex networks on different temporal and spatial scales [8]. Depending on the technique employed, neuroimaging can investigate the human brain’s functional and anatomical networks. Specifically, by use of positron emission tomography (PET) or functional MRI (fMRI), one can elucidate the neurophysiological dynamics of human brain networks; and using diffusion MRI, one can track the white-matter fibers passing through brain regions. This functional and anatomical information about brain networks has opened new opportunities to study the neural correlates of consciousness, and provided additional information concerning diagnosis, prognosis, and therapeutic effects in DOC patients.

Some excellent papers have reviewed the applications of neuroimaging in chronic DOCs from different points of view, for example, the clinical syndromes and pathophysiological mechanisms of DOCs [2] or various neuroimaging modalities [9, 10]. Here, we focus on brain network studies in chronic DOCs, including functional networks and anatomical networks. We also highlight some challenges and provide some perspectives on future work.

## Functional Brain Networks

Functional neuroimaging can measure the brain’s metabolic activity (for example, using PET), hemodynamic activity (for example, using fMRI or functional near-infrared spectroscopy (fNIRS)), and electrical activity (such as electroencephalography (EEG) or magnetoencephalography (MEG)). Brain functions in DOC patients can be investigated in the passive (i.e. after sensory stimulation), active (i.e. probing motor-independent signs of command-following), and task-free resting state. These elicited and spontaneous brain activities have provided informative windows to measure the impaired brain networks in DOC patients.

### Brain Network Studies of DOCs with PET

PET was one of the earliest functional neuroimaging methods to investigate DOCs, and it records brain metabolic processes *via* the emission of positrons from radioactively-labeled molecules. ^18^F-fluorodeoxyglucose (FDG) and H_2_^15^O are among the most widely-used labeled molecules in clinical practice and research. When the chosen molecule is FDG, the concentration of tracer indicates glucose uptake, indirectly representing local neural activity in a resting or task state; whereas H_2_^15^O, the density of which reflects blood flow, is usually used to detect activation during active and passive paradigms.

From the viewpoint of the total amount of metabolism within the brain, although DOC patients show a reduction of global metabolism to 40%–50% of normal values in the resting state [11], recovery of consciousness does not necessarily coincide with resumption of global metabolic activity [12]. This observation suggests that global brain metabolism is not a sensitive marker to trace the level of consciousness.

Voxel-based PET studies have indicated that the activities in particular brain regions or brain networks are more likely to reflect the level of consciousness. On the one hand, resting-state studies have demonstrated that DOC patients show decreased glucose uptake in a large-scale frontoparietal network, and the connections between the areas within the frontoparietal network and thalamic nuclei decline [13]. On the other hand, PET studies using passive auditory and noxious stimulation (i.e. electrical stimulation of the median nerve at the wrist) significantly activate the midbrain, contralateral thalamus, and primary sensory cortex; however, this cannot be processed by the association cortices in VS/UWS patients [14]. More importantly, a study with H_2_^15^O PET found that restoration of consciousness appears to be paralleled by the resumption of the functional relationship between the thalami and association cortices in VS/UWS patients [15]. In comparison, MCS patients show more metabolic preservation of the frontoparietal network. For example, auditory stimuli with emotional valence (i.e. the patient’s own name) induce a widespread activation, the pattern of which is comparable to that previously obtained in controls [16]; passive noxious stimuli elicit the activation of association areas related to pain-processing similarly to normal controls [17]. Together, these studies suggest the importance of the association cortices, rather than the primary sensory cortices, in the emergence of consciousness. The widespread frontoparietal network and its connections to thalamic nuclei are thought to be important for consciousness.

Recent studies have used machine-learning classifiers for the analysis of PET data, permitting calculation of the probability that individual patients are in VS/UWS or MCS, or have good outcome or not. These pilot studies at the single-patient level complement bedside examinations. Although PET activation studies recently appear to have been largely superseded by non-ionizing fMRI techniques, one study has suggested that cerebral PET, in comparison to task-activation fMRI, achieves higher accuracy in predicting the outcome for DOC patients [18].

### Brain Network Studies of DOCs with fMRI

fMRI measures brain activity by detecting the associated changes in blood flow. The primary form of fMRI uses blood-oxygen-level-dependent (BOLD) contrast [19]. Typically, it has the capacity to scan the entire brain with a spatial resolution of 2 mm–5 mm within 2 s–3 s. Therefore, fMRI can not only reveal the location of activity (functional segregation), but also probe the interactions between regions (functional integration). In short, the advantages of BOLD fMRI lie in its noninvasive nature, ever-increasing availability, relatively high spatiotemporal resolution, and capacity to demonstrate the entire network of areas, which have made it the mainstay of neuroimaging for functional brain network research [20]. A brief introduction to the principles of BOLD fMRI, and a review of various analysis methods, including functional connectivity, effective connectivity, and brain network construction and analysis, can be found in one paper [21].

#### Brain Network Studies of DOCs with Resting-State fMRI

Resting-state fMRI is particularly suitable for DOC patients because their interaction and/or application of possibly difficult experimental set-ups are not required. The method of functional connectivity estimates neural connectivity using the temporal correlation of pairs of voxels (or brain regions) in BOLD fMRI [22, 23]. It assumes that the more similar the time series between any given pair of voxels (or brain regions), the more likely it is that a functional connection exists between them. In addition, the functional connectivity is fast to compute, and does not require the scope of possible network models to be pre-specified or constrained. In the absence of a full understanding of the neural correlates of consciousness, functional connectivity analysis has computational advantages for network discovery and search.

Much progress has been made describing the damaged functional networks in DOC patients. The best-studied network is the default mode network (DMN), which includes the medial prefrontal cortex, the posterior midbrain regions, the medial temporal lobes, and the lateral parietal cortex. In healthy individuals, the DMN shows high levels of activity when no explicit task is performed [24, 25]. Although there are debates about the cognitive functions of the DMN [26–28], some investigators suggest that it directly contributes to internal awareness that is largely detached from the external world, including self-reflective thoughts and judgments, conceiving the mental states of others, and envisioning the future to make alternative decisions [29]. In particular, recent studies have found that the activity of the DMN is closely associated with specific states of consciousness, such as anesthesia [30] and sleep [31–33]. In DOCs, studies have shown that the resting-state functional connectivity within the DMN is decreased and proportional to the degree of consciousness impairment, from locked-in syndrome to MCS, VS, and coma patients [34]. Moreover, the reduced functional connectivity within the DMN, specifically between the medial prefrontal cortex and the posterior cingulate cortex, may predict the outcome for DOC patients [35].

Recently, more resting-state networks have been investigated in DOCs, such as executive control [36, 37], salience [38, 39], sensorimotor [40], auditory [41], visual [42], and subcortical networks [43]. It has been found that these networks or systems are also significantly impaired in DOC patients, and VS/UWS patients show more severe damage than MCS patients. Furthermore, studies based on machine-learning classification have found that these resting-state functional networks have a high capacity for separating patients into MCS and VS/UWS [41], and predicting their outcomes [37]. Notably, studies have suggested that the anti-correlation between the two diametrically opposed networks (i.e. DMN and executive control network) is one of the most crucial imaging features for predicting the outcomes of DOC patients [44].

#### Brain Network Studies of DOCs with Task-Activated fMRI

Sensory perceptions are related to consciousness. Although DOC patients cannot behaviorally respond to sensory stimuli, it is possible that stimuli can be perceived. In addition, “active” fMRI paradigms have been developed to probe for possible motor-independent signs of command-following. By detecting cortical responses to specific stimuli or commands, one can infer the level of consciousness of an individual DOC patient.

Many task-activated fMRI studies have reported near-normal high-level cortical activation in MCS and low-level activation in VS/UWS during auditory [45–48], visual [49, 50], noxious somatosensory [51, 52] and thermal [53] stimulation. Further, stimuli with emotional valence (i.e. the patient’s own name or a familiar picture) involve more brain areas than those responding to neutral stimuli [45, 50]. These studies have also reported that patients in MCS, compared to VS/UWS, have much more distributed activity and cortico-cortical connectivity in response to stimuli [45, 48, 54]. Patients, including MCS and VS/UWS, who exhibit high-level activation often show clinical signs of recovery at long-term follow-up [55].

The “active” fMRI paradigms require patients to follow commands and perform tasks, for example, motor (“imagine playing tennis”) [56], visuospatial (“imagine walking around in your house”) [56], or visual (“look at the face”) [49] domains. The presence of near-normal activity has been proposed as a marker for the recovery of consciousness [56]. This paradigm has been adapted into a yes/no answer communication system, allowing the DOC patient to communicate without using the traditional communication channels (motor or language) [11]. However, it is difficult to say whether this absence of brain activity following commands is the result of unconsciousness, so the “active” fMRI paradigms suffer from a high level of false-negative results.

Together, the resting-state and task-activated fMRI studies have confirmed previous PET results, and more importantly, they have suggested more complex and multifaceted alterations of the functional networks in DOC patients.

### Brain Network Studies of DOCs with fNIRS

fNIRS noninvasively monitors activity by measuring the absorption of near-infrared light through brain tissues [57]. Specifically, since the absorption spectra of oxyhemoglobin (HbO) and deoxyhemoglobin (HbR) in the 650–950 nm wavelength are different, it is possible for fNIRS to determine the relative concentration changes of HbO and HbR from diffusely-scattered light measurements, and then infer the brain activity [58–61]. fNIRS has a relatively superior spatial resolution (on the order of 1–2 cm) compared to EEG/MEG [62], and a relatively better temporal resolution (on the order of ms) compared to fMRI [63]. Most importantly, fNIRS is portable, silent, relatively low cost, easy to handle, and tolerant of movement artifacts and the measurement environment, making it possible to record long-time continuous and/or repeated measurements at the bedside of patients with DOCs [62]. fNIRS is not sensitive to metal implants such as deep brain or spinal cord stimulators, which makes it suitable for assessing real-time activity changes during neuromodulation therapy for patients with DOCs.

Researchers have used fNIRS to record activity in the bilateral motor cortex of DOC patients during a motor imagery task [64]. DOC patients had clearly lower HbO and HbR activity in the motor cortices during the task compared with the controls. Consistent with the controls, the evoked-HbO activity of the patients was significantly lateralized to the ipsilateral motor area. Although there was no significant difference between VS/UWS and MCS patients, this preliminary study demonstrated the feasibility of fNIRS for measuring brain activity in DOC patients.

More interestingly, by using a self-developed fNIRS system, researchers have measured real-time blood volume fluctuations in the prefrontal and occipital cortices during spinal cord stimulation (SCS) [65, 66]. They found that SCS induces significant cerebral blood volume changes, especially in the prefrontal cortex, even though the stimulation discharge period was very brief (30 s). Compared with long inter-stimulus intervals (3 or 5 min), a shorter interval (2 min) evoked more blood volume and had a long-term potential effect in the prefrontal cortex. This phenomenon was more evident in DOC patients with a favorable outcome.

Although fNIRS studies in DOCs are on the horizon, fNIRS has demonstrated potential and unique value for evaluating the activity of brain networks and therapeutic effects in DOCs.

### Brain Network Studies of DOC with Electrophysiology

The EEG records electrical activity by measuring voltage fluctuations resulting from ionic current within the neuron populations of the brain. Typically, it is noninvasive, with the electrodes placed on the scalp, while sometimes, invasive electrodes are used, as in electrocorticography (ECoG). Despite limited spatial resolution (a few centimeters), the EEG offers millisecond-range temporal resolution and directly reflects neuronal activity. In addition, it is one of the few mobile techniques available, so EEG continues to be a valuable tool for research and clinically. In DOCs, its applications generally focus either on the spectral content of the EEG or event-related potentials (ERPs). The former analyzes the type of neural oscillations that occur in the frequency domain of EEG signals. The latter investigates potential fluctuations time-locked to a stimulus onset. Recently, researchers have explored concurrent EEG responses to brain stimulation or manipulation, such as transcranial magnetic stimulation (TMS).

Based on recording ongoing EEG fluctuations, many types of information can be extracted including the power of fluctuations (local synchronization), functional and effective connectivity (interactions between areas), and topological characteristics of the brain network graphs (complexity measures in a network). Accumulating evidence has shown that these types of electrical activity in patients with DOCs are associated with behavioral consciousness [67–73], the metabolic demand of the brain [72], and clinical outcomes [71, 72, 74]. For example, compared with healthy participants, patients with DOC have increased delta power (0.1–3 Hz) [70, 72, 75] but decreased theta (4–7 Hz) and alpha (8–15 Hz) power [68–71, 76]. Furthermore, such changes in patients in VS/UWS are much more important than those for the patients in MCS. The EEG-derived functional and effective connectivity, as well as relevant network topography, are important for the diagnosis and prognosis of DOCs. In the alpha frequency band, patients with DOCs have consistently demonstrated decreased global-mean connectivity over the whole brain, reduced local and global network efficiency, and fewer hubs compared with healthy individuals [70, 76]. Studies have also found that the alpha-band connectivity for patients in VS/UWS is significantly lower than those in MCS, especially for the connectivity across distant sites [68, 72, 77, 78]. A recent remarkable brain networks study also reported that the participation coefficient (i.e. a metric indexing the presence of densely-interconnected central hubs) of the alpha-band network is strongly positively correlated with behavioral consciousness and brain metabolism [72]. In contrast, there is a consistently negative link between the functional brain network in the delta band and the level of consciousness [70, 72, 75] and clinical outcome of individual patients [72]. Gamma (32 Hz–100 Hz) synchrony has been demonstrated to be important for visual consciousness in healthy individuals [79]. Synchronization in the gamma-band frequency range is reportedly maintained in DOCs when top-down synchronization appears to be lost [80]. But it is rarely found to be correlated with the consciousness level of DOC patients, maybe due to the drastically reduced power of high-frequency neural activity in DOCs, including MCS and VS/UWS. Combining several EEG parameters may be a future direction of development. An automatic classification of patients’ state of consciousness has been proposed based on the combination of EEG parameters of low-frequency power, EEG complexity, and functional connectivity [71].

ERPs are time- and phase-locked activation components in response to particular events, which are extracted by averaging repeated stimulus-evoked EEG time series. For patients with DOCs, several passive (such as the auditory regularities violation task [71, 78, 81] and the local-global paradigm [82, 83]) and active (such as count names [67]) paradigms) have been used. Compared to the early ERP components such as N1 and P2, the late components like mismatch negativity, P300 or P3, and N400 are much more dependent on the participant’s consciousness state [84]. Some EPR components, especially P3, have been found to be specific potential markers of consciousness in individual patients [82, 83, 85, 86]. However, their diagnostic power in differentiating VS/UWS from MCS is suspected, due to a lack of sensitivity [87, 88].

Notably, a series of studies has recorded EEG data synchronously with both non-invasive brain stimulation (TMS [89], tDCS [90], and tACS [76]) and invasive brain stimulation (DBS [91] and SCS [92, 93]). In particular, TMS-EEG stimulates a specific brain area with a single TMS pulse, and monitors the local cortical reactivity as well as the rapid causal interactions among multiple groups of neurons thereafter [94, 95]. An index called the perturbational complexity index (PCI) has been proposed to quantify the complexity of local and distant brain responses to a TMS pulse [89]. It was demonstrated that PCI has a reliable ability to discriminate the level of consciousness in single individuals during wakefulness, sleep, and anesthesia, as well as in patients with DOCs (from VS/UWS to MCS to exit-MCS) [89, 96]. Its sensitivity in identifying patients with MCS is high, up to 94.7%. And 9 of 43 unresponsive VS/UWS patients with high values of PCI had a favorable clinical outcome at six months. Therefore, the complexity of TMS-EEG responses is a very attractive index for the diagnosis and prognosis of patients with DOC. In addition to TMS-EEG, synchronous recordings of EEG with other stimulation techniques have started to be used. Preliminary studies are mainly aimed to evaluate the feasibility of these neuromodulation protocols. For example, researchers found that oscillatory tDCS at 5 Hz on the cerebellum of patients with DOCs induced changes in cerebral frontoparietal networks [90]. In only MCS patients, these changes were positively correlated with a transient CRS-R amelioration after stimulation. Therefore, they proposed that tDCS cerebellar-cerebral connectivity modulation may be a useful approach in diagnosing chronic DOCs and ameliorating the level of consciousness.

Collectively, EEG-derived low-frequency power, ERPs, and functional connectivity/network analyses have demonstrated diagnostic and prognostic capabilities. In particular, the techniques of concurrent brain manipulation, including TMS and tDCS, and EEG imaging, show greatly encouraging clinical potential.

## Anatomical Brain Networks

To date, diffusion MRI (dMRI) is a unique non-invasive method for revealing the micro-geometry of nervous tissues and to explore white-matter fiber connectivity in the human brain. In DOCs, dMRI offers an opportunity to detect the damage to white matter and fiber tractography. White-matter abnormalities can be quantified by measures such as fractional anisotropy (FA), mean diffusivity (MD), or the apparent diffusion coefficient (ADC) in regions of interest or in a voxel-wise manner. In addition, using fiber-tracking techniques, one can carry out fiber tractography, and then investigate characteristics within fiber tracts or even in anatomical networks.

Numerous studies have identified white-matter abnormalities in DOCs using group-level analyses comparing varying levels of patients and healthy controls. Traumatic brain injury (TBI) is one of the etiologies of DOCs and has been investigated extensively using dMRI. The studies found that the corpus callosum [97–101] and internal capsule [97, 98] are the structures most vulnerable to TBI. In addition, the thalamus [97, 102] and whole-brain white matter [98, 99, 103] are also implicated in TBI. A meta-analysis demonstrated that the corpus callosum, and especially the splenium and body divisions, reliably reflect levels of consciousness, and FA measures correlate better with consciousness levels than MD (or ADC) in TBI [104]. Compared with a traumatic etiology, relatively few studies have explored the white-matter alterations in patients with anoxic brain injury. The findings include more distributed changes in white-matter bundles in anoxia [105, 106], including the cerebral peduncles, corpus callosum, thalamus, and brainstem. Studies have used fiber tractography to investigate white-matter abnormalities in DOCs. For example, researchers have investigated pathways connecting cortical regions within the DMN and pathways between DMN regions and the thalamus, and demonstrated impairments in these pathways correlated with various levels of consciousness [107].

In addition to identifying white-matter damage using group-level statistical analysis, there has been a recent interest in using machine-learning methods to automatically discriminate individuals with DOCs or predict the clinical outcome based on dMRI. Studies have reported 95% accuracy in discriminating between MCS and VS/UWS among 25 patients using MD maps of subcortical white matter and thalamic regions [108], and 81%–84% accuracy to correctly classify individuals across various levels of consciousness (VS, MCS–, and MCS+) using thalamocortical connectivity [109]. Personalized predictions of outcome have also been investigated. For example, a study examined 20 preselected white-matter tracts in 105 TBI patients from multiple centers and obtained an accuracy of 84% (95% sensitivity, 64% specificity) when identifying patients with a favorable outcome at 1 year follow-up [110]. Another study evaluated whole-brain voxel-wise FA in TBI patients and achieved an accuracy of 86% (86% sensitivity, 86% specificity) for early 1-year follow-up prognosis. In addition, FAs in the posterior corpus callosum, posterior limb of the internal capsule, inferior longitudinal fasciculus, and cerebral peduncle were specifically decreased in the unfavorable outcome group compared with the favorable outcome group [111]. Recently, a prediction analysis of TBI and non-TBI patients found that the radial diffusivity of the left superior cerebellar peduncle reached an accuracy of 87.5% at 3 months of follow-up [112].

As the only non-invasive and *in vivo* method available to study the integrity of white matter and anatomical networks, the dMRI has advanced our understanding of the neural substrate for the functional deficits identified in DOC patients, and shows potential for improving the diagnosis and prognosis at an individual patient level.

## Perspective

**Fig. 1:**
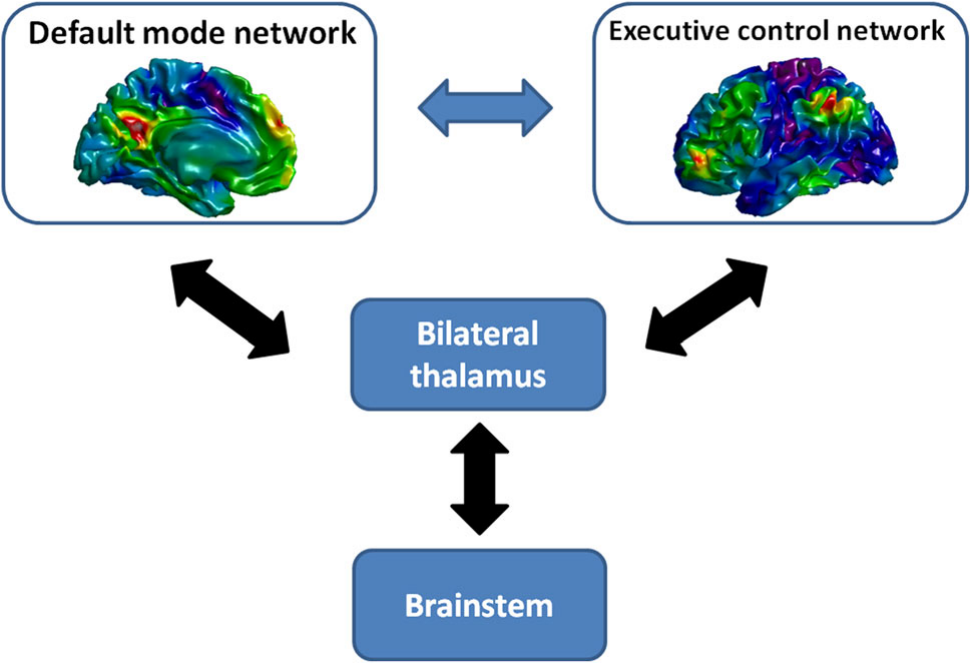
Brain networks and structures closely related to DOCs. The default mode network consists of medial prefrontal cortex, posterior midbrain regions, bilateral medial temporal lobes, and bilateral parietal cortex. The executive control network includes dorsal medial prefrontal cortex, bilateral anterior prefrontal cortex and bilateral superior parietal cortex. The double-sided arrows represent the reciprocating functional links between the relevant cortical networks and thalamus and brainstem.

Taken together, brain imaging studies have suggested complex and multifaceted alterations of brain anatomical and functional networks in DOC patients. In particular, the connectivities within the DMN and thalamus, and the anti-correlations between the DMN and the executive control network could be crucial for tracing the level of consciousness in the DOC (Fig. 1). These brain areas and their connections have shown potential for informing the diagnosis and prognosis in DOC.

**Fig. 2:**
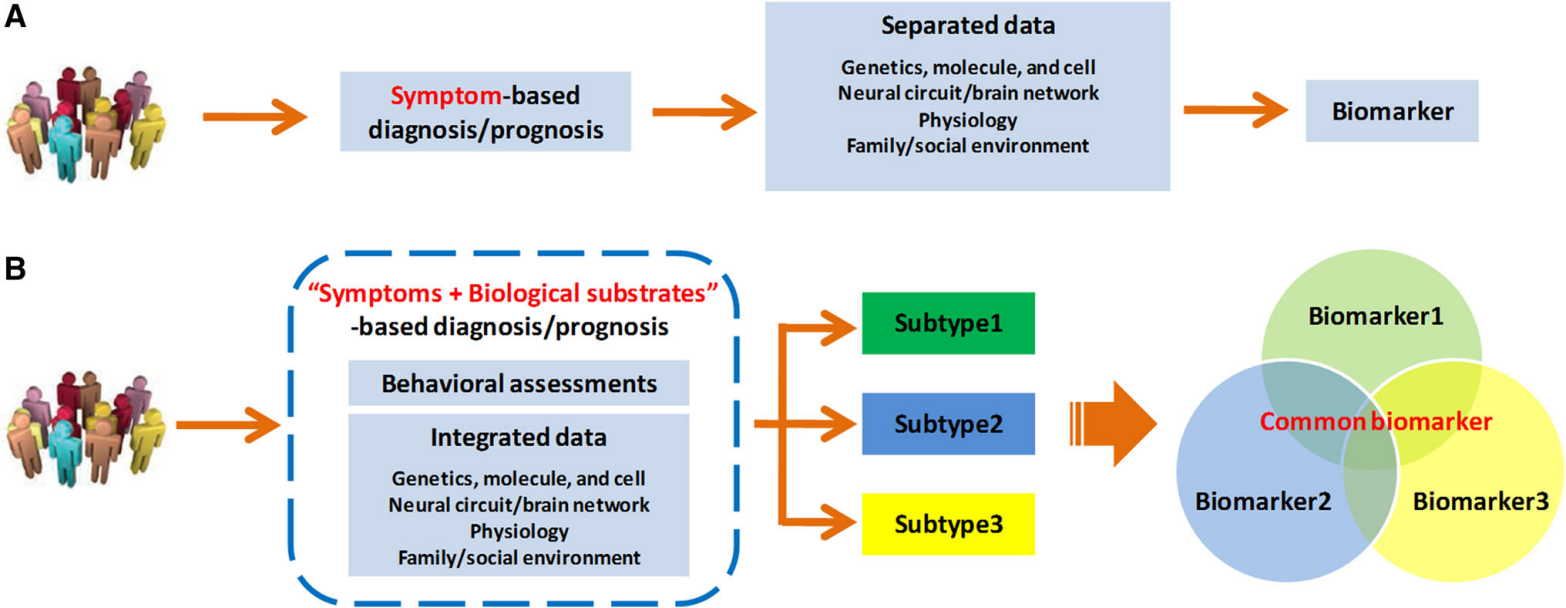
Regular (**A**) and proposed (**B**) research tracks for DOCs. In the regular research paradigm (**A**), researchers have used only behavior-based assessments as the gold standard for dividing DOC patients into different groups and then making comparisons between groups, or classifying DOC patients with different clinical categories. The studies are separated and thus the findings are not converging. In our proposal as in (**B**), the diagnosis and prognosis of DOCs integrate behavioral evidence with biological substrates that come from approaches such as genetics, molecular biology, and brain imaging. More importantly, it is necessary to deconstruct the mixed DOC populations into biologically validated subgroups, that is “subtypes”, and then seek discriminatory biomarkers for DOCs.

Despite many advances, the physiological disturbances and the circuit/network differences in DOC studies have fallen short of biomarker standards. The diagnosis and prognosis for DOCs remain restricted to subjective symptoms and observable signs. Researchers still use behavior-based assessments alone as the gold standard for dividing DOC patients into different groups, or classifying DOC patients with different clinical categories. The regular research paradigm is shown in Fig. 2A. In fact, DOCs are a heterogeneous mixture of different diseases. The DOC patients share impaired consciousness in common, but this can have many different causes, be associated with several neuropathological processes, and show different severities. For example, the CRS-R is composed of 23 items distributed in six subscales assessing different functions (auditory, visual, motor, oromotor/verbal, communication, and arousal). Patients are diagnosed as MCS when they demonstrate at least one of the eleven items (consistent movement to command; reproducible movement to command; object recognition; object localization: reaching; visual pursuit; fixation; automatic motor response; object manipulation; localization to noxious stimulation; intelligible verbalization; nonfunctional: intentional); emergence from MCS is signaled by at least one of the two items: functional communication and object use. That allows for several hundred unique combinations of changes in the remaining brain functions. The association between clinical symptoms and the underlying biological substrates is inconsistent and variable at the individual level even when diagnosed as MCS. Therefore, it is increasingly necessary to deconstruct current DOC groups into biologically validated subgroups, that is “subtypes”, for understanding the various aspects of dysfunction and improving the accuracy with which patients are categorized and treated (Fig. 2B).

Consciousness comes from the brain. The human brain can be studied as a hierarchy of distinct but tightly-integrated levels of organization: from gene, protein, synapse, neuron, and neural circuit, to brain area, pathway, and the whole brain. Although advances have been made, the findings mostly focus on a single level, which can only reflect limited aspects of how the brain is formed and how it works. There is no doubt that any single technique has its own advantages and limitations, and cannot address all issues concerning DOCs. Table 1 lists some typical brain imaging/ manipulating techniques and the relevant findings in DOC. More and more evidence suggests that combinations of different technologies could generate more data than simply pooling the findings from a single technology. For example, equipment that combines fNIRS and EEG technology can detect neurophysiological activity and oxygen fluctuations simultaneously, which has great potential for finding unpredictable results in patients with DOCs. Therefore, to understand how the brain contributes to consciousness and unravel the neuropathology of unconsciousness, it is urgent to integrate a variety of techniques, methods, and models, and to merge fragmented findings into a uniform research framework or platform. Here, we propose that the “Brainnetome” (Brain-net-ome) is a notable framework to comprehensively explore the brain’s anatomical and functional networks at various spatiotemporal scales [113]. In particular, the “Brainnetome” atlas [114] is an *in vivo* brain map, with fine-grained functional subregions and detailed anatomical and functional connection patterns for each area, which can help researchers to more accurately locate impaired brain activity and connectivity in DOCs.

**Table 1:** Some typical brain imaging/manipulating techniques and relevant findings in DOC.

Techniques	Usages	Characteristics	Findings in DOC
*Imaging techniques*			
PET	To detect local metabolic processes or blood flow changes in brain during the resting or task state	1. Temporal resolution: seconds/minutes 2. Spatial resolution: millimeters	1. Global brain metabolism could not be a sensitive marker to trace the level of consciousness 2. Frontoparietal network and their connections to thalamus nuclei are important for emergence of consciousness
fMRI	To detect brain activity by measuring BOLD change, and explore functional connections between brain areas	1. Temporal resolution: seconds 2. Spatial resolution: millimeters 3. Utilize the synchronization of BOLD signals as functional connectivity or effective connectivity to represent connections 4. Carry on brain functional network analysis based on multiple regions of interest within the whole brain	1. Several resting state networks are disrupted in DOC 2. Functional connections within the DMN and between the DMN and ECN may be crucial for diagnosis and prognosis in the DOC
fNIRS	To detect brain activity by measuring changes of attenuation of near-infrared through one’s cortex, and explore functional connections	1. Temporal resolution: 1/10 second 2. Spatial resolution: centimeters 3. Carry on brain functional network analysis based on signals of optodes 4. Utilize the synchronization of changes of concentrations of oxyhemoglobin or deoxyhemoglobin as functional connectivity or effective connectivity to represent connections	1. fNIRS has unique value for evaluating the activity of brain network and therapeutic effects in the DOC
EEG	To record electrical activity in the brain, and explore neural oscillations/interactions in the frequency domain or potential fluctuations time locked to a stimulus onset	1. Temporal resolution: milliseconds 2. Spatial resolution: centimeters 3. Carry on brain functional network analysis based on either signals of electrodes or inverse mapped signals on the brain 4. Utilize the synchronization of fluctuations of electrical fields as functional connectivity or effective connectivity to represent connections	1. Increased delta power but decreased theta and alpha power in the DOC 2. Several functional brain network indexes in delta and alpha bands show correlations with the level of consciousness. 3. MMN, P3, and N400 can provide useful information about level of consciousness in the DOC
dMRI	To measure the diffusion of water along axon, and estimate the major fiber tracts between the brain areas	1. The only noninvasive method for quantifying the white matter connectivity *in vivo* 2. Spatial resolution: millimeters 3. Utilize some characteristics of fiber tracts to represent connections 4. Carry on brain anatomical network analysis based on multiple regions of interest within the whole brain	1. The DOCs with different etiologies demonstrate distinct distributions of impaired white matters 2. Fibers connecting cortical regions within DMN and between DMN regions and thalamus are correlated to levels of consciousness
*Concurrent manipulating and imaging techniques*			
TMS-EEG	To explore the changes of brain network dynamics, and to further probe the degree of complex brain activity supporting consciousness	1. Utilize TMS to send a single pulse of magnetic energy to brain 2. Monitor the induced electrical activity in the underlying cortex by a high-density EEG 3. Estimate the complexity of the induced EEGs	1. The perturbation complexity index is proposed to differentiate if patients are conscious or unconscious
tDCS/tACS-EEG	To establish the causal link between different brain areas, and to explore the changes of brain network dynamics	1. Stimulate the particular brain areas 2. Monitor the induced electrical activity in the underlying cortex by EEG 3. Analyze EEG-derived functional and effective connectivity, as well as relevant brain network dynamics	1. The tDCS/tACS may be a useful approach to improving diagnosis and ameliorating the level of consciousness
TMS-fMRI	To establish the causal link between different brain regions and even the dynamics of the whole brain, including the subcortical areas	1. Special non-ferrous TMS coils are used to stimulate the brain 2. fMRI is typically acquired continuously, with a need to avoid coincidences with the TMS pulse	1. The TMS-evoked regional activity and network connectivity are disrupted in DOC
DBS/SCS-fNIRS	To evaluate the dynamics of brain network and therapeutic effects	1. Electrical stimulation on brain deep nuclei or spinal cord 2. fNIRS assesses real-time brain activity changes during DBS or SCS	1. DBS/SCS could induce significant cerebral blood volume changes in the cortex

DOC, disorders of consciousness; PET, positron emission tomography; MRI, magnetic resonance imaging; fMRI, functional MRI; BOLD, blood-oxygen-level-dependent contrast; DMN, default mode network; ECN, executive control network; fNIRS, functional near-infrared spectroscopy; EEG, electroencephalography; MMN, mismatch negativity; dMRI, diffusion MRI; TMS, transcranial magnetic stimulation; tDCS, transcranial direct current stimulation; tACS, transcranial alternating current stimulation; DBS, deep brain stimulation; SCS, spinal cord stimulation

Further, techniques for neuroscience can be roughly divided into two classes based on their functions. One class is techniques that measure brain structure and/or function while individuals are in particular states or diseases. The above reviewed PET, fMRI, fNIRS, EEG, and dMRI fall into this category. Another class is manipulation techniques that cause the brain structure and/or function to change and consequently influence behavior, for example, DBS, SCS, nerve stimulation, TMS, tDCS/tACS, and transcranial focused ultrasound stimulation. By combining the manipulation techniques and brain imaging, one can measure the brain activity driven by stimulated neurons in local brain areas or even across the whole brain. These methods complement each other and provide different views of brain networks. More importantly, they allow mapping of the causal functional connectivity between brain areas and further probing of the dynamics of functional networks in DOCs. For example, TMS-EEG can establish a perturbation complex index of brain networks for DOCs [115, 116]. SCS-fNIRS reflects real-time blood volume fluctuations in the cortex during SCS [66]. Recently, some more compatible (for example, MRI-compatible) techniques have been developed. We believe that this kind of “concurrent manipulation + observation” technique will reveal more basic findings and clinical applications [117, 118].

The convergence of diverse disciplines, especially biology, medicine, and informatics, is dramatically changing brain research. The development of new imaging and data analysis technologies has revolutionized our ability to measure and understand the brain and its diseases. The advances in imaging technology have made it possible to noninvasively and *in vivo* image the human brain at unprecedented spatiotemporal resolution, including manifesting the shapes and sizes of brain areas, mapping the fibers linking different brain areas, and elucidating the networks and pathways responsible for specific functions. The deluge of these complex and heterogeneous biological and medical data poses significant challenges for the informatics community. Novel data modeling and analysis techniques, especially artificial intelligence techniques, have demonstrated great improvements in extracting, comparing, searching, and managing biological and medical data. Therefore, how to organize so many researchers with diverse disciplines is a major challenge for the future of DOC research.
